# GEAR 2.0 ‐ Communication and Shared Decision Making

**DOI:** 10.1002/alz.091839

**Published:** 2025-01-09

**Authors:** Christopher R Carpenter

**Affiliations:** ^1^ Mayo Clinic, Rochester, MN USA

## Abstract

Aging adults worldwide are presenting to the emergency department with acute and subacute illness and injury confounded by often unrecognized cognitive impairment, including dementia and delirium. Conveying medical information and weighing various diagnostic and therapeutic approaches during times of emergency is difficult for all aging adults. In adult ED populations *without* dementia, communication is imperfect with incomplete recollection of test results, presumptive diagnoses, prescriptions, and follow‐up recommendations. Communication strategies striving to improve ED nurse/physician‐to‐patient information exchange such as TeachBack are ineffective in non‐dementia populations and untested in subsets with dementia or their care partners. Increasingly, healthcare disparities associated with dementia detection and interventions are acknowledged. For example, limited health literacy is one source of disparity, interacts with the severity of dementia, is one communication barrier, and yet is not routinely evaluated in ED settings. Shared Decision Making is an evolving approach to engage patients and families in complex medical conversations, but emergency medicine shared decision making research often excludes individuals with dementia by design. This session will explore theoretical opportunities to improve medical decision‐making during times of emergency for persons living with dementia and their care partners.

